# Evaluating the efficacy and mechanism of metformin targets on reducing Alzheimer’s disease risk in the general population: a Mendelian randomisation study

**DOI:** 10.1007/s00125-022-05743-0

**Published:** 2022-07-29

**Authors:** Jie Zheng, Min Xu, Venexia Walker, Jinqiu Yuan, Roxanna Korologou-Linden, Jamie Robinson, Peiyuan Huang, Stephen Burgess, Shiu Lun Au Yeung, Shan Luo, Michael V. Holmes, George Davey Smith, Guang Ning, Weiqing Wang, Tom R. Gaunt, Yufang Bi

**Affiliations:** 1 Department of Endocrine and Metabolic Diseases, Shanghai Institute of Endocrine and Metabolic Diseases, Ruijin Hospital, Shanghai Jiao Tong University School of Medicine, Shanghai, China; 2 Shanghai National Clinical Research Center for Metabolic Diseases, Key Laboratory for Endocrine and Metabolic Diseases of the National Health Commission of the PR China, Shanghai Key Laboratory for Endocrine Tumor, State Key Laboratory of Medical Genomics, Ruijin Hospital, Shanghai Jiao Tong University School of Medicine, Shanghai, China; 3 MRC Integrative Epidemiology Unit (IEU), Bristol Medical School, University of Bristol, Oakfield House, Oakfield Grove, Bristol, UK; 4 Clinical Research Center, The Seventh Affiliated Hospital, Sun Yat-sen University, Shenzhen, Guangdong China; 5 Center for Digestive Disease, The Seventh Affiliated Hospital, Sun Yat-sen University, Shenzhen, Guangdong China; 6 Guangzhou Women and Children Medical Center, Guangzhou, Guangdong China; 7 Division of Epidemiology, the JC School of Public Health & Primary Care, the Chinese University of Hong Kong, Hong Kong, Hong Kong; 8 MRC Biostatistics Unit, Cambridge Institute of Public Health, Cambridge, UK; 9 Cardiovascular Epidemiology Unit, Department of Public Health and Primary Care, University of Cambridge, Cambridge, UK; 10 School of Public Health, Li Ka Shing Faculty of Medicine, The University of Hong Kong, Pokfulam, Hong Kong, SAR China; 11 Medical Research Council Population Health Research Unit, University of Oxford, Oxford, UK; 12 Clinical Trial Service Unit & Epidemiological Studies Unit, Nuffield Department of Population Health, University of Oxford, Oxford, UK; 13 National Institute for Health Research, Oxford Biomedical Research Centre, Oxford University Hospital, Oxford, UK; 14 NIHR Biomedical Research Centre at the University Hospitals Bristol NHS Foundation Trust and the University of Bristol, Bristol, UK

**Keywords:** Alzheimer’s disease, Brain expression, Cognitive function, Dementia, Mendelian randomisation, Metformin targets, Mitochondrial function

## Abstract

**Aims/hypothesis:**

Metformin use has been associated with reduced incidence of dementia in diabetic individuals in observational studies. However, the causality between the two in the general population is unclear. This study uses Mendelian randomisation (MR) to investigate the causal effect of metformin targets on Alzheimer’s disease and potential causal mechanisms in the brain linking the two.

**Methods:**

Genetic proxies for the effects of metformin drug targets were identified as variants in the gene for the corresponding target that associated with HbA_1c_ level (*N*=344,182) and expression level of the corresponding gene (*N*≤31,684). The cognitive outcomes were derived from genome-wide association studies comprising 527,138 middle-aged Europeans, including 71,880 with Alzheimer’s disease or Alzheimer’s disease-by-proxy. MR estimates representing lifelong metformin use on Alzheimer’s disease and cognitive function in the general population were generated. Effect of expression level of 22 metformin-related genes in brain cortex (*N*=6601 donors) on Alzheimer’s disease was further estimated.

**Results:**

Genetically proxied metformin use, equivalent to a 6.75 mmol/mol (1.09%) reduction on HbA_1c_, was associated with 4% lower odds of Alzheimer’s disease (OR 0.96 [95% CI 0.95, 0.98], *p*=1.06×10^−4^) in non-diabetic individuals. One metformin target, mitochondrial complex 1 (MCI), showed a robust effect on Alzheimer’s disease (OR 0.88, *p*=4.73×10^−4^) that was independent of AMP-activated protein kinase. MR of expression in brain cortex tissue showed that decreased MCI-related gene (*NDUFA2*) expression was associated with lower Alzheimer’s disease risk (OR 0.95, *p*=4.64×10^−4^) and favourable cognitive function.

**Conclusions/interpretation:**

Metformin use may cause reduced Alzheimer’s disease risk in the general population. Mitochondrial function and the *NDUFA2* gene are plausible mechanisms of action in dementia protection.

**Graphical abstract:**

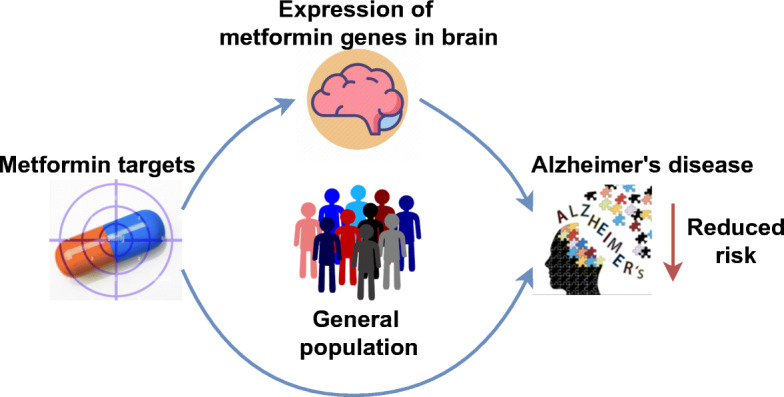

**Supplementary Information:**

The online version of this article (10.1007/s00125-022-05743-0) contains peer-reviewed but unedited supplementary material..

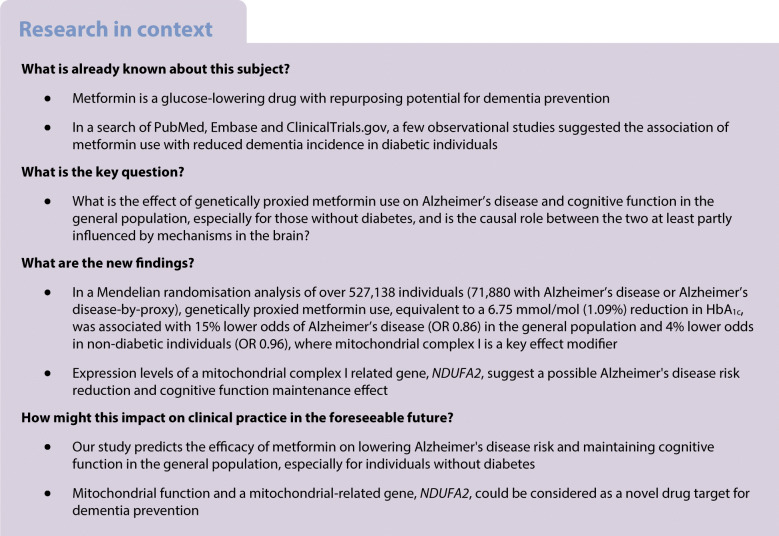



## Introduction

Metformin is an efficient first-line glucose-lowering therapy to manage hyperglycaemia in diabetic individuals. It is a desirable drug-repurposing candidate to improve ageing-related disorders, both dependent on and beyond glycaemic control [1]. Dementia has been reported to be associated with treatment status of diabetes [2]. Recent observational studies further suggest the association of metformin use with incident dementia in diabetic individuals [3]. Large-scale trials such as Targeting Aging with Metformin (TAME) [4] include incident dementia as one of their primary endpoints. However, these trials are still in their early stages and it will be several years before the trial evidence is released. Therefore, the causal role of metformin on dementia is under-studied, especially in people without diabetes [5]. In addition, metformin has a beneficial effect on the heart, kidney and brain via different biological pathways [6]. Whether metformin’s beneficial effect on dementia is due to glucose control or at least partly due to other mechanisms in the brain is worth further investigation. A study that accurately estimates the causal effect and mechanism of metformin on dementia in the general population will provide timely evidence to guide future clinical trials of metformin.

Mendelian randomisation (MR) is a genetic epidemiology method that uses genetic variants as predictors to estimate the causal effect of a modifiable exposure on an outcome [7, 8]. The approach has previously been used to evaluate the effect of glycaemic phenotypes and metformin on cardiovascular diseases and cancers [9–14]. The genetic data utilised in MR analysis are typically generated in large-scale biobanks and/or consortia, representing exposure/outcome status in the general population. This is therefore an ideal approach by which to estimate metformin’s effect on dementia in the general population.

The overall effect of metformin is influenced by multiple pharmacological targets, including AMP-activated protein kinase (AMPK) [5], mitochondrial complex 1 (MCI) [5], mitochondrial glycerol 3 (MG3) [15], growth differentiation factor 15 (GDF15) [16] and glucagon-like peptide-1 (GLP1)/glucagon (GCG) [5]. Their effects therefore need to be considered together. Moreover, novel molecular phenotypes such as gene expression data [17] and new methods such as genetic colocalisation [18] have been used widely to identify tissue-specific causal genes for complex diseases, which can be used to investigate the biological mechanisms involved in metformin’s action on dementia.

The objective of this study was to estimate the causal effect of metformin on Alzheimer’s disease (or Alzheimer’s disease-by-proxy) and cognitive function in a general European population using MR. Through this approach, we further investigated whether expression of metformin-related genes in the brain showed an effect on Alzheimer’s disease or Alzheimer’s disease-by-proxy, with the findings potentially guiding drug repurposing of metformin and novel drug target identification for dementia prevention.

## Methods

### Study design and participants

**Fig. 1:**
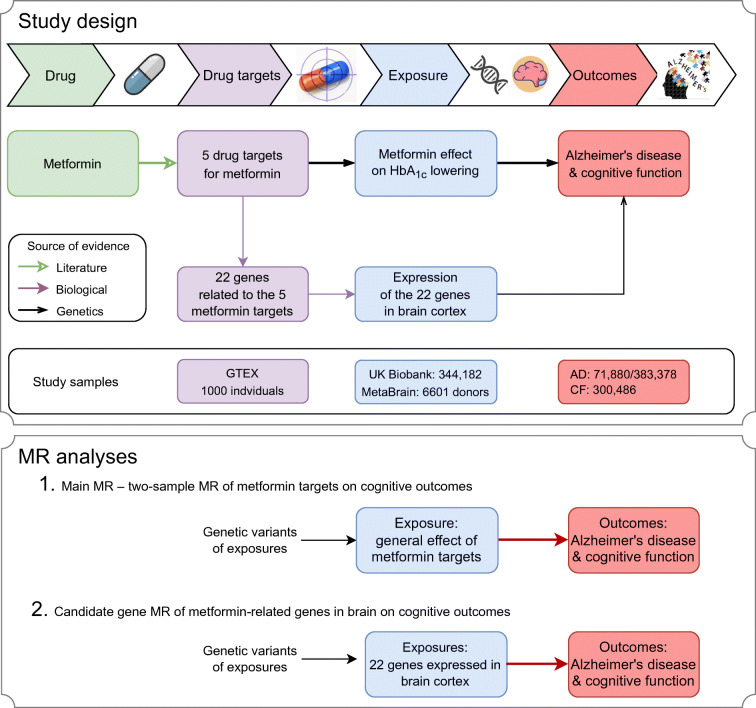
Diagram of the study design. This MR study aims to identify the causal relationships between metformin (drug), five metformin-related targets (drug targets), general metformin effects (exposure), expression of 22 metformin-related genes (exposures), and Alzheimer’s disease/cognitive function (outcomes). Three levels of evidence were used to construct the causal atlas, including literature and biological and genetic evidence. AD, Alzheimer’s disease; CF, cognitive function

Figure 1 illustrates the design of this study. We aimed to understand the causal role of metformin (drug) on two cognitive outcomes, Alzheimer’s disease or Alzheimer’s disease-by-proxy (*N* clinically diagnosed cases=24,087, *N* proxy cases=47,793, *N* controls=383,378; we treated Alzheimer’s disease and Alzheimer’s disease-by-proxy as cases in this study, with the caution that Alzheimer’s disease-by-proxy was an approximation based on parental diagnoses [19]) and cognitive function [20] (*N*=300,486). Since MR by definition proxies specific drug target effects rather than the general drug effect (potentially on multiple proteins/pathways), we searched for drug targets of metformin in the literature (from inception to 1 March 2021), and identified five targets: AMPK [5], MCI [5], MG3 [15], GDF15 [16] and GLP1/GCG [5]. We then identified genes involved in the action of these five targets using the ChEMBL database [21] (from inception to 1 March 2021; electronic supplementary material [ESM] Fig. 1). Genetic proxies for the effects of the five metformin drug targets were identified, from which we selected 32 genetic variants near each of the 22 metformin genes that associated with both the glycaemic biomarker HbA_1c_ (*N*=344,182 UK Biobank individuals, 5.3% clinical diagnosed diabetic individuals) and the expression level of the corresponding gene (*N*≤31,684; 49 available human tissues; data from GTEX [22], eQTLGen [23] and Zheng et al [24]; ESM Fig. 2). The exposures were defined as metformin’s glucose-lowering effect via the five targets. In addition, to understand whether metformin-related genes may influence Alzheimer’s disease or Alzheimer’s disease-by-proxy and cognitive function in the brain, we selected brain expression levels of 22 genes involved in metformin’s action (*N*=6601 brain donors from the MetaBrain consortium [17]) as a second set of exposures. To identify the causal links between the exposures and outcomes, we integrated two state-of-the-art genetic epidemiology approaches, MR and genetic colocalisation [18, 24–26], and developed an analysis pipeline to obtain reliable causal estimates in a general population of middle-aged Europeans.

### Selection and validation of genetic predictors of metformin’s effects

As illustrated in ESM Fig. 1, the selection of genetic predictors of metformin’s glucose-lowering effect involved three steps: (1) map metformin to five of its pharmacological targets (AMPK, MCI, MG3, GDF15 and GLP1/GCG); (2) map the five metformin targets to their related genes (ESM Table 1); (3) map the metformin-related genes to related genetic variants (for more details, see ESM Method 1). The genetic variants for each metformin target involved in the selection process are listed in ESM Tables 2–6.

To select valid genetic predictors that proxy the glucose-lowering effect of metformin, we applied MR and genetic colocalisation methods [18, 24] to filter genetic variants (or their proxies with pairwise squared correlation [*r*^2^] to nearby variants over 0.8; such correlation is described as linkage disequilibrium in genetics) to those with evidence to support a shared genetic association signal between changing the expression level of a metformin-related gene and changing HbA_1c_ level (more details are given in ESM Fig. 1 and ESM Method 1). Since metformin exerts actions in multiple tissues, we used all 49 tissues that were available from the expression studies [22–24]. After validation, the genetic predictors for each target were generated, with effects quantified as the HbA_1c_-lowering effect of the target. In total, 32 genetic variants within 22 genes were selected as predictors for metformin’s HbA_1c_-lowering effect (ESM Table 7).

### Selection and validation of genetic predictors of metformin-related genes in the brain

Some of the metformin-related genes are expressed in the brain [17]. Based on this, we hypothesised that changes in the expression of metformin-related genes in the brain may influence cognitive outcomes. To identify candidate genes in the brain, we searched the genetic variants associated with the brain expression levels of the 22 metformin genes and used them as potential mediators for the mediation analysis. The MetaBrain consortium meta-analysed expression quantitative trait locus (eQTL) data of human genes in brain tissues. The term eQTL here refers to genetic variants associated with the expression level of a gene. The most statistically powerful eQTL dataset was obtained in brain cortex, in which gene expression levels of 6601 brain donors were measured [17]. In this study, we searched for eQTLs of the 22 metformin-related genes in brain cortex (ESM Table 8A). To select the best genetic predictors, we picked eQTLs with the lowest *p* value that also had a pairwise linkage disequilibrium *r*^2^ (squared correlation) less than 0.001 to nearby eQTLs as an indication of selecting independent predictors.

### Outcomes

We selected cognitive outcomes that are currently undergoing metformin trials using information from the ChEMBL [21] and ClinicalTrials.gov databases. This identified two cognitive outcomes: cognitive function and Alzheimer’s disease. The genetic associations for these two outcomes were extracted from recent genome-wide association studies, with 24,087 clinically diagnosed Alzheimer’s disease cases, 47,793 Alzheimer’s disease-by-proxy cases (Alzheimer’s disease reported in a parent), 383,378 controls [19] and 300,486 individuals with cognitive function records [20]; these studies are among the largest available for these outcomes to date (ESM Table 8B).

### Statistical analyses

Figure 1 presents the two main analyses conducted in this study: (1) the main MR analysis estimating the effect of metformin targets on cognitive outcomes; and (2) the candidate gene analysis estimating the effects of 22 metformin genes in the brain on cognitive outcomes.

For the main MR analysis, we estimated the general effect of metformin on the two cognitive outcomes: cognitive function and Alzheimer’s disease (Fig. 1). To achieve this, we first estimated the target-specific effect of the five metformin targets (AMPK, MCI, MG3, GDF15 and GLP1/GCG) using MR. If a genetic predictor was missing in the outcome data, a genetic variant with high pairwise correlation (*r*^2^>0.8) was used to proxy the missing predictor. The general metformin effect was estimated using the 32 genetic variants close to the 22 metformin-related genes. The Cochran’s *Q* test was applied to estimate the heterogeneity across genetic predictors.

For the candidate gene MR analysis, we estimated the putative causal effects of brain expression levels of metformin-related genes on the two cognitive outcomes (Fig. 1). The 22 metformin-related genes were considered as candidate genes for this analysis. Among the 22 genes, 20 obtained well-powered genetic predictors for their expression level in brain cortex (ESM Table 9A). Cognitive function and Alzheimer’s disease were considered as outcomes. Given the limited number of predictors for each gene, we applied the Wald ratio and inverse variance weighted approaches followed by genetic colocalisation to increase reliability of the findings.

To further estimate whether the effect of metformin targets on Alzheimer’s disease and cognitive function occurs via HbA_1c_ lowering or other mechanisms, we conducted a sensitivity analysis of circulating HbA_1c_ on Alzheimer’s disease and cognitive function using 99 genetic predictors derived from the MAGIC consortium [27], irrespective of genomic position of genetic variants (ESM Table 9B). Due to the potential influence of erythrocyte phenotypes on HbA_1c_ levels, we excluded HbA_1c_ gene variants associated with erythrocyte distribution and/or erythrocyte count (ESM Table 9C) and ran MR against Alzheimer’s disease and cognitive function. As a further validation, we also estimated the effect of genetic liability to type 2 diabetes on Alzheimer’s disease and cognitive function (ESM Table 9D).

### Follow-up MR analysis

First, to validate the findings using different MR methods, we conducted a one-sample MR using individual-level data from 360,347 unrelated Europeans in the UK Biobank (for further details, see ESM Method 2). Second, inhibition of MCI will result in the activation of AMPK [5]. Therefore, we investigated the combined and independent effects of MCI and AMPK targets on cognitive function using a factorial MR approach (for further details, see ESM Method 2).

### Triangulation of genetic and observational evidence

We triangulated the genetic evidence from MR and pharmacoepidemiology studies from the literature (from inception to 1 March 2021) to seek positive controls by which we might approximate a scaling mechanism of clinical trial effects using genetic data. We searched PubMed from inception to 1 March 2021 for meta-analyses evaluating the effects of metformin on Alzheimer’s disease. The literature search identified one meta-analysis of observational studies [28], and we used this for our triangulation analysis. We rescaled the observational and MR estimates to OR of Alzheimer’s disease risk and compared the effect estimates of the two different approaches (for further details, see ESM Method 3).

### Test for MR assumptions

MR relies on three core assumptions (ESM Fig. 3): (1) relevance (i.e. the genetic predictors are robustly associated with the exposure, HbA_1c_); (2) exchangeability (i.e. the association of genetic predictors with Alzheimer’s disease and cognitive function is not confounded); and (3) exclusion restriction (i.e. the effect of the genetic predictors on Alzheimer’s disease and cognitive function are only through the exposure). This study reports findings based on the STROBE-MR guidelines (ESM Method 4) [29], testing the three MR assumptions using the following approaches.

The relevance assumption was validated using two approaches. First, MR and colocalisation analyses [18, 24] were conducted between the expression level of the metformin-related genes and HbA_1c_ to select genetic variants robustly associated with both phenotypes (ESM Fig. 1). Second, the strength of the genetic predictors of each tested metformin target were estimated using the proportion of variance in each exposure explained by the predictor (*R*^2^) and *F* statistics. An *F* statistic above 10 is indicative of evidence against weak instrument bias [30].

The exchangeability assumption was tested by performing genetic colocalisation analysis between HbA_1c_ (exposure) and Alzheimer’s disease/cognitive function (outcomes). This approach aims to distinguish real gene–disease associations from spurious associations created due to confounding by correlated genetic variants [24]. A colocalisation probability (*p*) of 70% or more between the gene and outcome phenotype was used as evidence of colocalisation and recorded as ‘colocalised’. The rest were recorded as ‘not colocalised’.

The exclusion restriction assumption was tested using the following sensitivity approaches: MR Egger regression [31]; weighted median analysis [32]; and mode estimator analysis [33]. A single-variant MR comparison was carried out to examine whether MR estimates were driven by a single influential variant in drug target proxies. All these sensitivity methods were conducted using functions implemented in the TwoSampleMR package [25].

For all MR analyses, a conservative Bonferroni-corrected threshold was used to account for multiple testing.

## Results

### Strength of the genetic predictors of the metformin targets and genes

We first estimated the instrument strength, which indicates statistical power of the genetic predictors of metformin targets and genes. All exposures had strong instruments (*F* statistics over common threshold of 10; ESM Tables 7 and 9), except for the GLP1/GCG target (*F* statistic = 3.9). We kept all the exposures and mediators in the analyses but with the understanding that the genetic predictors of GLP1/GCG could be influenced by weak instrument bias.

### Effect of metformin on Alzheimer’s disease and cognitive function

**Fig. 2:**
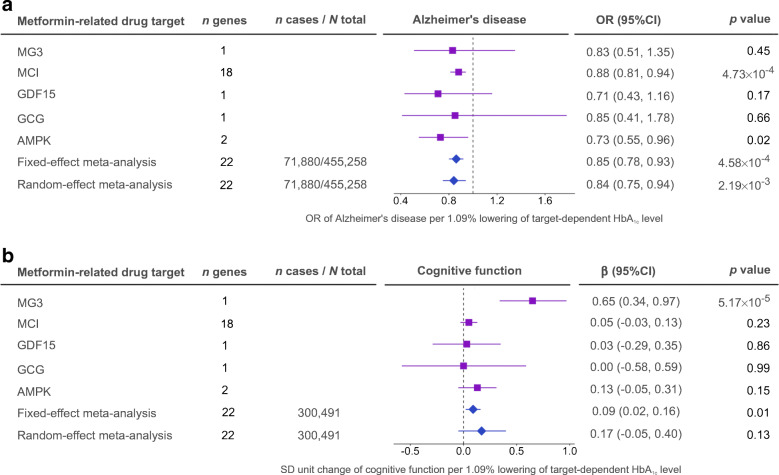
MR analysis of metformin’s effects on Alzheimer’s disease and cognitive function. The OR (**a**) and the SD unit change (**b**) are shown for the five metformin-related targets. Purple squares represent the effects of the five targets on the two outcomes; blue diamonds represent the fixed-effect and random-effect meta-analyses estimating the general effect across the five targets. One SD unit lowering of HbA_1c_ refers to 6.75 mmol/mol (1.09%) reduction in HbA_1c_

For the main MR analysis (Fig. 1), we estimated the general effects of metformin on the two cognitive outcomes (two tests, Bonferroni-corrected *p*=0.025). The MR analysis suggested a general effect of metformin targets on reducing Alzheimer’s disease risk (OR 0.85 [95% CI 0.78, 0.93], *p*=4.58×10^−4^; Fig. 2a and ESM Table 10A) and maintaining cognitive function in the general population (β 0.09 [95% CI 0.02, 0.16], *p*=0.01; Fig. 2b). The sensitivity analysis using genetic predictors and outcome data derived from individuals without diabetes still suggested a protective effect on Alzheimer’s disease risk in non-diabetic individuals (OR 0.96 [95% CI 0.95, 0.98], *p*=1.06×10^−4^; ESM Table 10B). The MCI-specific effect of metformin was associated with reduced Alzheimer’s disease risk (OR 0.88, *p*=4.73×10^−4^; Fig. 2a); this was the strongest effect among the five targets. The heterogeneity test of each metformin target showed little evidence to support heterogeneous effects across the five targets (ESM Table 10A). Other sensitivity analyses suggested that these effects were robust to various MR assumptions (ESM Fig. 4, ESM Tables 11, 12).

In addition, using 99 HbA_1c_ instruments, we observed little evidence of an effect of genetically predicted circulating HbA_1c_ on Alzheimer’s disease or cognitive function (ESM Table 10C), implying that the effect of metformin targets on Alzheimer’s disease is likely to be through a glycaemia-independent mechanism. Using 45 HbA_1c_ instruments excluding genetic variants associated with erythrocyte phenotypes or using 118 type 2 diabetes instruments, we found that neither genetically predicted circulating HbA_1c_ nor genetic liability to type 2 diabetes were likely to be associated with Alzheimer’s disease or cognitive function (ESM Table 10C).

As a positive control, we replicated the known effect of metformin on reducing type 2 diabetes risk using both fixed-effect and random-effect inverse variance weighted models (OR 0.68 [95% CI 0.50, 0.91], *p*=9.7×10^−3^; ESM Table 10D), which validated the reliability of our genetic predictors and MR approaches.

### Effect of gene expression levels in the brain on Alzheimer’s disease and cognitive function

**Fig. 3:**
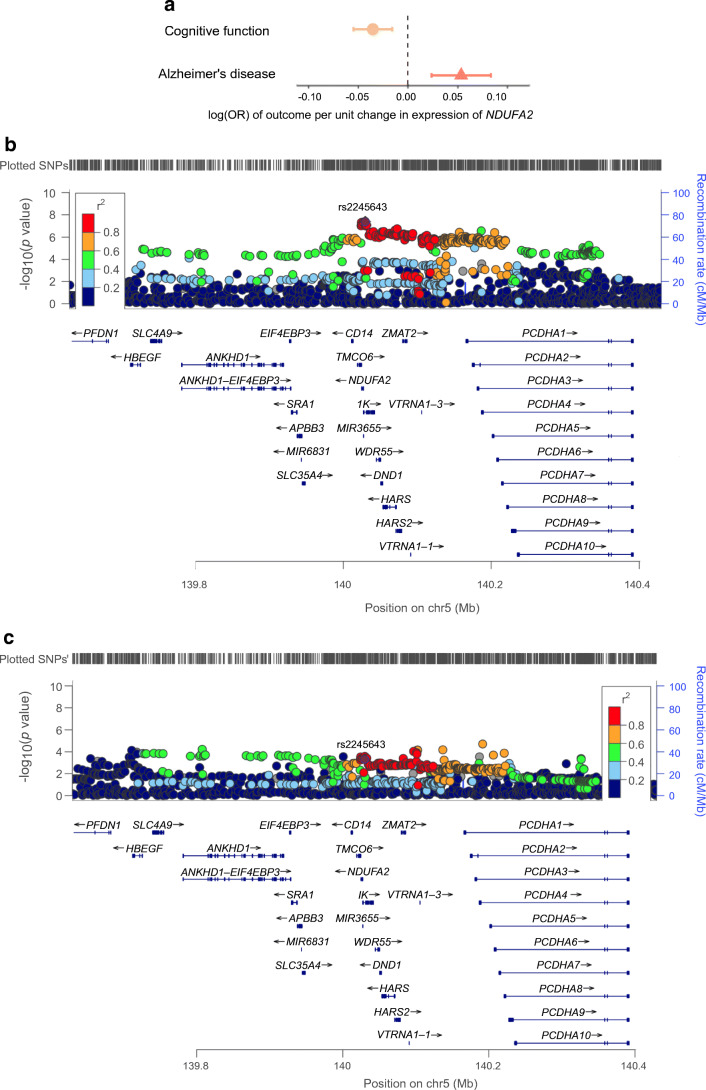
MR effects of *NDUFA2* expression in brain cortex on Alzheimer’s disease and cognitive function. (**a**) MR estimates, presented as log(OR) of outcome per unit change in expression of *NDUFA2*. Bars show the 95% CI of the effect estimates. (**b**) Regional plot of *NDUFA2* expression in the *cis*-acting *NDUFA2* region. (**c**) Regional plot of Alzheimer’s disease in the *NDUFA2* region. Result of other genes are listed in ESM Table 13. One unit refers to 1 SD of the expression level change of *NDUFA2*

We further investigated the putative causal effects of metformin-related genes on AD and cognitive function (Fig. 1). Due to data availability, we were able to conduct MR analysis of 17 genes on Alzheimer’s disease and 13 genes on cognitive function (in total 30 tests, Bonferroni-corrected *p*=1.67×10^−3^). As shown in Fig. 3, decreased expression level of an MCI-related gene, *NDUFA2*, in brain cortex was associated with reduced Alzheimer’s disease risk (OR 0.95, *p*=4.64×10^−4^) and maintained cognitive function (β 0.04, *p*=4.09×10^−4^). The colocalisation analysis provided robust evidence to support this putative causal effect (colocalisation *p*=83% and 82%, respectively; ESM Table 13). Increased expression of an AMPK-related gene, *PRKAA1*, showed evidence of an effect on reducing Alzheimer’s disease risk (OR 0.95, *p*=2.36×10^−3^), slightly under the Bonferroni-corrected threshold. A summary of the main MR and candidate gene MR results are presented in ESM Fig. 5.

### Follow-up analyses and triangulation of metformin effects on cognitive function

**Fig. 4:**
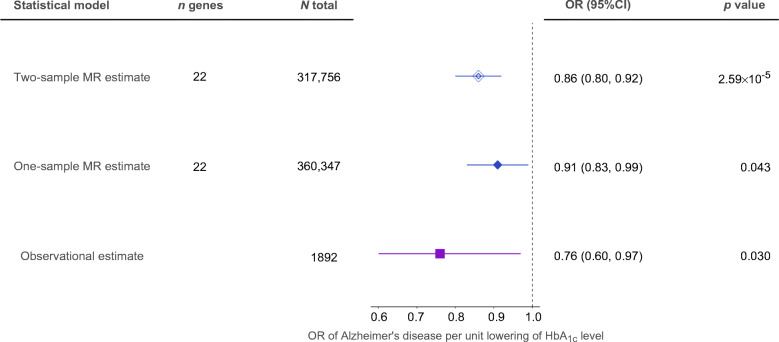
Triangulation of observational estimate from meta-analysis, one-sample MR evidence and two-sample MR evidence for metformin effect on cognitive function. Data are presented as SD unit increase of cognitive function per 6.75 mmol/mol (1.09%) reduction of HbA_1c_ via metformin use. One unit refers to 1 SD change in cognitive function. Bars show the 95% CI of the effect estimates

We conducted two follow-up analyses to validate the effect of metformin on cognitive function. First, one-sample MR confirmed the effect of metformin on cognitive function using individual-level UK Biobank genotype and phenotype data (weights to build the genetic score are presented in ESM Table 14; results are presented in ESM Table 15A). Second, we investigated the independent effects of MCI and AMPK on cognitive function. The results suggested that the MCI-specific effect of metformin was associated with cognitive function independently of the AMPK effect (ESM Table 15B). In addition, we triangulated the existing pharmacoepidemiology evidence from the literature with the genetic evidence we obtained from this study. One-sample MR, two-sample MR and observational estimates suggested that a reduction in cognitive decline may occur with metformin use, with the effect sizes comparable across different methods (Fig. 4).

## Discussion

Genetics has shown value in predicting drug trial success in previous studies [24, 34]. In this study, we observed that lifelong naturally randomised genetically proxied metformin use leads to a 15% reduction of Alzheimer’s disease risk and maintained cognitive function in the general population, of which 4% reduction of Alzheimer’s disease risk was observed in the non-diabetic individuals. Genetic effects on MCI predicted a beneficial effect on Alzheimer’s disease that is independent of AMPK. Our candidate gene analysis suggested a causal role for the expression level of an MCI-related gene, *NDUFA2*, on Alzheimer’s disease and cognitive function, with this effect likely to be localised in brain. Collectively, these findings provide key evidence to guide future clinical trials of metformin and prioritise metformin-related genes as novel targets for dementia prevention.

Metformin is proposed to be beneficial for cognitive outcomes. In observational studies, metformin showed association with reduced dementia incidence in diabetic individuals [3, 6]. To date, some early-stage trials of metformin in dementia prevention are in progress (ClinicalTrials.gov registration no. NCT04098666, NCT03861767). Large-scale trials such as TAME are still in their early stages [4]. It will be several years before these trials release their results. Our results provide genetic evidence to support the causal effect of metformin on reduced Alzheimer’s disease risk in the general population (of which up to 10% are diabetic in Western nations). This finding provides novel evidence to extend the generalisability of metformin’s effects on dementia prevention to non-diabetic individuals, addressing a key gap in the current literature evidence. Our findings also promote metformin repurposing as a potential dementia prevention strategy for future trial design.

Metformin has a clear role in inhibiting MCI of the respiratory chain. This action prevents mitochondrial ATP production and activates AMPK. Both will result in the inhibition of gluconeogenesis [5]. In this study, our MR results suggested a mitochondrial-specific causal effect on reducing Alzheimer’s disease risk, independent of the AMPK effect. This highlights the value of lowering glucose via metformin use on dementia prevention. In this study, we also found that inhibition of expression of an MCI-related gene, *NDUFA2*, in brain cortex was linked with reduced Alzheimer’s disease risk and maintained cognitive function, providing human genetics evidence to ease such concerns. The *NDUFA2* gene is part of MCI and may regulate complex I activity. Previous genetic studies reported this gene to be associated with brain white matter [35]. An observational study suggested *NDUFA2* as a biomarker for Alzheimer’s disease [36]. This implies that the prioritised gene, *NDUFA2*, can be considered as a potential drug target for dementia prevention, both dependent on and independent of metformin’s action.

By reviewing existing clinical trial and/or MR studies of medication treatment and/or drug repurposing for Alzheimer’s disease (from inception to 1 March 2021), we found some evidence to support the role of liraglutide (a GLP-1 inhibitor) and most of the antihypertensive drugs on preventing/delaying cognitive impairment [37–41]. The level of prevention of dulaglutide (another GLP-1 inhibitor) was similar to the effect estimate we observed on metformin targets (14%) [37]. We also found that cholinesterase inhibitors and *N*-methyl-d-aspartate receptor antagonist were the two most widely used types of medication for dementia management [42, 43], as recommended by the current National Institute for Health and Care Excellence (NICE) guidance for people with Alzheimer’s disease (https://www.nice.org.uk/guidance/cg42). With evidence to support the effect of metformin in Alzheimer’s disease prevention in future clinical trials, we consider metformin as an additional therapy for those who cannot tolerate marketed drugs such as cholinesterase inhibitors, and for high-risk individuals with diabetes, insulin resistance and obesity.

Our study has several strengths. By definition, MR estimates the effects of a drug’s targets and/or genes related to a drug’s action rather than the direct effect of the drug (e.g. metformin use) on the diseases. In this study, we developed a novel genetic epidemiology strategy to estimate the target-specific effect and meta-analyse these effects to obtain the general metformin effect on Alzheimer’s disease. This strategy extends the scale of ‘drug target’ MR to multi-target drugs. Such a strategy also boosts the power of the analysis by including genetic predictors from multiple targets of the same drug.

Our study also has limitations. First, by the nature of MR, our estimates represent the average linear causal effects across the general population. With the development of novel approaches such as non-linear MR [44], we will investigate the dose–response causal effect of metformin on dementia prevention in the near future. Second, the MR analysis of molecular phenotypes (e.g. expression levels of genes) uses a small number of genetic predictors and this can lead to concerns regarding weak instrument bias. However, since we used gene expression data from over 6000 brain donors, our genetic predictors of metformin-related genes obtained good instrument strength. Third, the biology of metformin is still only partly understood. There is a possibility that our study missed targets and genes that are still under investigation or are difficult to target using existing genetic tools (e.g. gut microbiota [45]). However, we selected metformin targets by systematically reviewing the literature and relevant databases and this review is the most systematic MR study of metformin targets performed to date. We hope, with development of updated genetic studies (e.g. of microbiota) and new genetic tools, our analysis pipeline could be extended to any newly identified metformin targets in the future. Fourth, some of our instruments were associated with blood cell phenotypes, which is a limitation of using HbA_1c_ as proxy. However, it is one of the best glucose measurements over a few months and probably the most stable representative to proxy effects of metformin targets. Fifth, MR represents lifetime manipulation of metformin targets, whereas the treatment would certainly not be started either at the fetal stage or during most of the early life. This is a key consideration for drugs/drug targets for Alzheimer’s disease prevention, since some clinical trials on targets with genetic evidence, such as β-site amyloid precursor protein cleaving enzyme 1 (BACE1) inhibitors, failed to show efficacy after billions of US dollars of investments in pharma [46]. One potential reason for this failure is that the treatment was started too late, with the damage having already been done earlier in the disease development.

Our study represents a comprehensive investigation of metformin using genetics, providing robust evidence to support the causal effect of metformin on reducing Alzheimer’s disease risk in the general European population. We reveal the independent role of inhibition of MCI on reducing Alzheimer’s disease risk and identified a mitochondrial-related gene, *NDUFA2*, as a key mediator in the brain. These findings provide evidence to support repurposing metformin and prioritising a metformin-related target/gene for dementia prevention in the general population.

## Supplementary Information

ESM 1 (PDF 1013 kb)

ESM 2 (XLSX 544 kb)

## Data Availability

The data, analytical methods and study materials will be made available to other researchers for the purposes of reproducing the results. In more detail, the genetic association data of the selected risk factors are available in ESM Tables. The GWAS summary statistics for the 21 primary outcomes are available from the IEU OpenGWAS database (https://gwas.mrcieu.ac.uk/). The UK Biobank received ethical approval from the Research Ethics Committee (REC reference for the UK Biobank is 11/NW/0382). The analytical script of the MR analyses conducted in this study is available via the GitHub repository of the ‘TwoSampleMR’ R package (https://github.com/MRCIEU/TwoSampleMR/).

## Abbreviations

AMPK: AMP-activated protein kinase
eQTL: Expression quantitative trait locus
GCG: Glucagon
GDF15: Growth differentiation factor 15
GLP1: Glucagon-like peptide-1
MCI: Mitochondrial complex 1
MG3: Mitochondrial glycerol 3
MR: Mendelian randomisation
TAME: Targeting Aging with Metformin
