# Supplementary material for: Evaluating the efficacy and mechanism of metformin targets on reducing Alzheimer’s disease risk in the general population: a Mendelian randomisation study
Source: Diabetologia. 2022 Jul 29;65(10):1664–75. doi: 10.1007/s00125-022-05743-0 (PMC9477943; doi:10.1007/s00125-022-05743-0)
Supplement: Supplementary file 1 — (PDF 1013 kb) [file 125_2022_5743_MOESM1_ESM.pdf]

ESM Method 1. Selection and validation of genetic predictors

Selection of genetic predictors

As demonstrated in **ESM Fig. 1**, we used a three steps approach to the selection of genetic predictors of metformin targets and genes. We first mapped metformin to five pharmacologically active targets by searching drug substance information from literatures: AMPK, MCI, MG3, GDF15 and GLP1/GCG. Second, we mapped the five targets to related genes using information from CHEMBL[1] and DrugBank[2]. Using this approach, we identified 68 genes that related to metformin actions (MCI: 58 genes, MG3: 1 gene, AMPK: 7 genes, GDF15: 1 gene and GCG: 1 gene, **ESM Table 1**). Third, for the 68 metformin related genes, we systematically scanned genetic variants associated with the expression levels of these genes using data from seven recent GWAS studies of genes level in 49 human tissues and proteins in plasma[3,4,5,6,7,8,9]. A p-value cut-off of 0.05 was used to select genetic variants associated with the expression levels of the 68 metformin genes. This p-value threshold was applied to maximise total prediction of the functions of the five targets.

This step mapped 68 metformin-related genes to 2,021 genetic variants. The number of genetic variants selected for each metformin target were: MCI: 1,631 variants (**ESM Table 2**), MG3: 48 variants (**ESM Table 3**), AMPK: 292 variants (**ESM Table 4**), GDF15: 43 variants (**ESM Table 5**) and GCG: 6 variants (**ESM Table 6**).

Validation of genetic predictors

We further applied MR and genetic colocalization methods[10,11] to filter genetic variants for the target-based analysis. In more details, the variant-expression associations were used as exposure, the variant-glucose associations were used as outcome to estimate the expression-glucose association via the tested variant in this validation analysis (**ESM Fig. 2**). Genetic colocalization was further applied to confirm the expression-glucose associations via the tested variants were not biased by nearby genetic variants that correlated with the tested variants (in other words, the tested variant showed dummy effect on glucose due to correlation with another variant, rather than the tested variant having a true effect on glucose). For genetic variants with evidence of an effect on glycemic traits, we selected those with the lowest P value that had a pairwise squared correlation (r^2) less than 0.001 as this indicates little correlation among the selected genetic variants). European population specific correlations among variants were estimated from the 1000 Genomes Project (phase 3) implemented in the two-sample MR package[15,12].

To quantify the statistical power of the analysis, we estimated the strength of the genetic predictors of each tested exposure using F-statistics. The strength of the predictors mimicking HbA_{1c} effects were well-powered and used as exposures for the target-based analysis (**ESM Table 7**). For predictors mimicking other glycemic traits (including fasting glucose, 2-hour glucose, HOMA-B and HOMA-IR), the statistical power of the genetic predictors was very low (average F-statistic<1), and therefore were not included in further analysis.

ESM Method 2. Sensitivity analysis results validating main MR results

One-sample Mendelian randomization validating the two-sample MR analysis results

We conducted one-sample MR to validate the effect of metformin on cognitive function. For genetic predictors of metformin, we looked up the genetic association information of HbA_{1c} lowering (**ESM Table 14**) and used them as the weights to build the genetic score for metformin's HbA_{1c} lowering effect. The individual level cognitive function data were used as outcome for the one-sample MR. Cognitive function was defined using the same selection criteria described in a recent cognitive function GWAS[14], in which the 13 multiple-choice questions that assessed verbal and numerical reasoning (noted as 'fluid' cognitive test in the UK Biobank, data-Field 20016) were used. We restricted analysis to 360,347 unrelated European samples to control for the influence of population structure and relatedness.

Estimation of independent effect of MCI and AMPK on BMI and DBP

In addition, we investigated the independent (or interactive) effects of MCI and AMPK targets on cognitive function. We applied a multivariable regression model to estimate the interactive effect, which was described as "interactions between two interventions" in a previous paper [15]. In the model, the genetic scores of MCI and AMPK targets were built up using the genetic association information of HbA_{1c} lowering listed in **ESM Table 14**. Then, cognitive function was regressed against the genetic scores of MCI and AMPK as well as the product of the two scores in the two multivariable models presented below:

Cognitive function ~ MCI_score + AMPK_score + (MCI_score x AMPK_score) + age + sex + chip + 10 PCs

Where 10 PCs refers to the first 10 principal components of the genetic data in UK Biobank, which were used to control for population structure.

ESM Method 3. Literature search and triangulation of MR and trial evidence.

For the MR association of metformin on cognitive function, we searched PubMed (from inception to March 1, 2021) for meta-analyses evaluating the effects of metformin on cognitive function. The search was perfumed using the following combined text and MeSH terms: 'metformin' and 'cognitive function', with no restriction on language (see search strategy in the **Appendix box**). If more than one meta-analysis was identified, we only included the latest study, the one based on randomized controlled trials, or the one reported appropriate pooled estimated effect that could be compared with the MR results.

Search	Query	Results
#1	Search: **metformin [Text Word]**	24,012
#2	Search: **metformin [MeSH Terms]**	14,200
#3	Search: **(cognitive function [MeSH Terms]) OR (cognitive function [Title/Abstract])**	193,658
#4	Search: **(meta-analysis [Title/Abstract]) OR (meta-analysis [MeSH Terms])**	182,535
#5	Search: **(systematic review [Title/Abstract]) OR (systematic review [MeSH Terms])**	183,812
#6	Search: **#1 OR #2**	24,012
#7	Search: **#3 OR #4 OR #5**	288,388
#8	Search: **#6 AND #7**	172

Appendix box. Search strategy for the literature review.

The literature search identified 172 records, of which 40 non-meta-analyses were excluded after reviewing titles and abstracts. Three studies were induced after full-text screening. This included one meta-analysis of observational study for cognitive function [16]. This is a new area that ongoing trials such as TAME would like to study, but there is only one pilot trial in a small number of individuals available in the literature at this time. Therefore, the triangulation of cognitive function was carried out using meta-analyses of observational evidence instead. The genetic evidence was extracted from the two-sample MR of HbA_{1c} lowering via MG3 on cognitive function, where the genetic association information of the outcomes was obtained from large-scale GWAS of cognitive function (sample size equal to 317,756).

We further rescaled the observational and MR estimates to odds ratio to make the genetic evidence more comparable to the observational evidence[17,18]. However, we still recognised that genetic predictors for exposures represent lifelong durations of exposure and the nature of the outcome-exposure relationship (e.g. whether it is cumulative)[19]. This makes direct comparisons of estimates derived from a genetic predictor to a observational association challenging.

ESM Method 4. STROBE-MR checklist

Item	Complete/location
**1. Title and Abstract:** "Mendelian randomization" is named both in the title and the abstract	Complete
**Introduction**	
**2. Background:** Explain the scientific background and rationale for the reported study. Is causality between exposure and outcome plausible? Justify why MR is a helpful method to address the study question.	Concept of Mendelian randomization and specific request for drug target Mendelian randomization were explained in the second paragraph of the introduction.
**3. Objectives:** State specific objectives clearly, including pre-specified causal hypotheses (if any).	The causal question has been stated in the third paragraph of the introduction.
**Methods**	
**4. Study design and data sources:** Present key elements of study design early in the paper. Consider including a table listing sources of data for all phases of the study. For each data source contributing to the analysis, describe the following:<br>a) Describe the study design and the underlying population from which it was drawn. Describe also the setting, locations, and relevant dates, including periods of recruitment, exposure, follow-up, and data collection, if available.<br>b) Give the eligibility criteria, and the sources and methods of selection of participants.<br>c) Explain how the analyzed sample size was arrived at.<br>d) Describe measurement, quality and selection of genetic variants.<br>e) For each exposure, outcome and other relevant variables, describe methods of assessment and, in the case of diseases, the diagnostic criteria used.<br>f) Provide details of ethics committee approval and participant informed consent, if relevant.	All necessary information about the GWAS studies been used in this study have been described in the method section and ESM Table 8.<br><br>The genetic predictor selection process has been described in in the Methods section “Selection and validation for genetic predictors of metformin effects” and in ESM Method 1.<br><br>Ethics approval and informed consent info in the "Study Populations" section of the method section.
**5. Assumptions:** Explicitly state assumptions for the main analysis (e.g. relevance, exclusion, independence, homogeneity) as well assumptions for any additional or sensitivity analysis.	The Mendelian randomization assumptions have been described in method section “Test for Mendelian randomization assumptions” and ESM Fig. 4.
**6. Statistical methods main analysis** Describe statistical methods and statistics used.<br>a) Describe how quantitative variables were handled in the analyses (i.e., scale, units, model).<br>b) Describe the process for identifying genetic variants and weights to be included in the analyses (i.e, independence and model). Consider a flow diagram.<br>c) Describe the MR estimator, e.g. two-stage least squares, Wald ratio, and related statistics.	(a) Described in methods and Figure 2, 3, 4.<br>b) Described in the section " Selection and validation for genetic predictors of metformin effects" within Methods.<br>c) Described in the section " Statistical analyses" within Methods<br>d) Described in the section " Statistical analyses" within Methods<br>e) Described in the section " Statistical analyses" within Methods

Detail the included covariates and, in case of two-sample MR, whether the same covariate set was used for adjustment in the two samples.<br>d) Explain how missing data were addressed.<br>e) If applicable, say how multiple testing was dealt with.	
**7. Assessment of assumptions: Describe any methods used to assess the assumptions or justify their validity.**	We have drafted a specific section “Test for Mendelian randomization assumptions” in the Methods, which explained how we deal with each of the Mendelian randomization assumption in this study.
**8. Sensitivity analyses:** Describe any sensitivity analyses or additional analyses performed.	The Mendelian randomization sensitivity analyses have been listed in " Test for Mendelian randomization assumptions" section of the Method. The follow-up sensitivity analyses been applied was listed in “Follow-up analyses” and ESM Method 2.
**9. Software and pre-registration**<br><br>a) Name statistical software and package(s), including version and settings used.<br>b) State whether the study protocol and details were pre-registered (as well as when and where).	a) All statistical software and settings used are described in the “Data and materials availability” section.<br>b) The analysis plan was described in the " Study design and participants” section of the Methods and Figure 1.
**Results**	
**10. Descriptive data**<br>a) Report the numbers of individuals at each stage of included studies and reasons for exclusion. Consider use of a flow-diagram.<br>b) Report summary statistics for phenotypic exposure(s), outcome(s) and other relevant variables (e.g. means, standard deviations, proportions).<br>c) If the data sources include meta-analyses of previous studies, provide the number of studies, their reported ancestry, if available, and assessments of heterogeneity across these studies. Consider using a supplementary table for each data source.<br>d) For two-sample Mendelian randomization:<br>i. Provide information on the similarity of the genetic variant-exposure associations between the exposure and outcome samples.<br>ii. Provide information on extent of sample overlap between the exposure and outcome data sources.	a) Information is given in the " Study design and participants" section of the Methods and Figure 1.<br>b) We listed the detailed information of the summary statistics for our instruments in ESM Tables 1-9. Summary statistics are also available from each GWAS as described in the "Outcomes" section of the Methods.<br>c) We give this information in ESM Table 4.<br>d) We provide this information in the "Study design and participants" section of the Method.
**11. Main results**<br><br>a) Report the associations between genetic variant and exposure, and between genetic variant and outcome, preferably on an interpretable (e.g. comparing 25th and 75th percentile of allele count or genetic risk score, if individual-level data available).<br>b) Report causal effect estimate between exposure and outcome, and the measures of uncertainty from the MR analysis. Use an intuitive scale, such as odds ratio, or relative	a) Genetic exposure associations have been reported in ESM Table 2-9.<br>b) The causal effect estimates between exposures, mediators and outcomes were listed in Figure 2, 3, 4, ESM Table 10-14 and S16<br>Our results were presented in terms of odds ratio and confidence intervals throughout the results section for binary outcomes and as beta coefficient for quantitative outcomes<br>d) We visualize results using three sets of forest plot in Figures 2, 3 and 4, S5 and S6, and as a scatter plot of SNP effects on exposures versus outcomes in ESM Fig. 5.

risk, per standard deviation difference.<br>c) If relevant, consider translating estimates of relative risk into absolute risk for a meaningful time-period.<br>d) Consider any plots to visualize results (e.g. forest plot, scatterplot of associations between genetic variants and outcome versus between genetic variants and exposure).	
**12. Assessment of assumptions**<br><br>a) Assess the validity of the assumptions.<br>b) Report any additional statistics (e.g., assessments of heterogeneity, such as I2, Q statistic).	a) We assess the validity using sensitivity analyses, MR Egger regression, weighted median approach and mode estimate approach and signal variant Mendelian randomization approach. Results were presented in Results<br>b) We discuss the use of Cochran's Q, Rucker's Q and I^2 statistic in the Results.
**13. Sensitivity and additional analyses**<br>a) Use sensitivity analyses to assess the robustness of the main results to violations of the assumptions.<br>b) Report results from other sensitivity analyses (e.g., replication study with different dataset, analyses of subgroups, validation of instrument(s), simulations, etc.).<br>c) Report any assessment of direction of causality (e.g., bidirectional MR).<br>d) When relevant, report and compare with estimates from non-MR analyses.<br>e) Consider any additional plots to visualize results (e.g., leave-one-out analyses).	a) we reported the use of genetic colocalization as additional approach to test for Mendelian randomization for the causal gene analysis of expression of metformin related genes on dementia and cognitive function.<br>b) validation of instruments were reported in "Strength of the genetic predictors of the metformin targets and genes" in Results.<br>d) A factorial Mendelian randomization was applied to estimate the independent effect of AMPK and MCI on cognitive function in section "Follow-up analyses and triangulation of metformin effects on cognitive function".<br>The results comparing one-sample MR, two-sample MR and observational correlation of metformin use on cognitive function was showed in section "Follow-up analyses and triangulation of metformin effects on cognitive function".<br>e) the scatter plot and single variant Mendelian randomization plot was presented in ESM Fig. 5.
**Discussion**	
**14. Key results**	Discussion paragraph 1
**15. Limitations**<br>Discuss limitations of the study, taking into account the validity of the MR assumptions, other sources of potential bias, and imprecision. Discuss both direction and magnitude of any potential bias, and any efforts to address them.	Discussion paragraph 5
**16. Interpretations**<br>a) Give a cautious overall interpretation of results considering objectives and limitations.<br>Compare with results from other relevant studies.<br>b) Discuss underlying biological mechanisms that could be modelled by using the genetic variants to assess the relationship between the exposure and the outcome.<br>c) Discuss whether the results have clinical or policy relevance, and whether interventions could have the same size effect.	a) Interpretation: Discussion paragraphs 1, 2, 3, 4, 5, 6;<br>Comparison with other studies: Discussion paragraphs 2, 3, 4.<br>b) Discussion paragraph 3<br>c) Discussion paragraph 1, 2, 6.

**17.**	**Generalizability:**	We have discussed the potential caveats in terms of generalizability of our findings in the fifth paragraph of the Discussion section.
**18.**	**Funding:**	We have reported all sources of funding in the "Acknowledgements" section.
**19.**	**Data and data sharing:**	We have provided the link/approach to access genetic data used in this study in the " Data and materials availability" section. The software and scripts been used in this study was listed in the same section.
**20.**	**Conflicts of Interest:**	All authors have declared conflicts of interest (none reported).

References for ESM Methods

1 Mendez D, Gaulton A, Bento AP, *et al.* ChEMBL: towards direct deposition of bioassay data. *Nucleic Acids Res* 2019; **47**: D930–40.

2 Wishart DS, Knox C, Guo AC, *et al.* DrugBank: a comprehensive resource for in silico drug discovery and exploration. *Nucleic Acids Res* 2006; **34**: D668-72.

3 Sun BB, Maranville JC, Peters JE, *et al.* Genomic atlas of the human plasma proteome. *Nature* 2018; **558**: 73–9.

4 Folkersen L, Fauman E, Sabater-Lleal M, *et al.* Mapping of 79 loci for 83 plasma protein biomarkers in cardiovascular disease. *PLoS Genet* 2017; **13**: e1006706.

5 Suhre K, Arnold M, Bhagwat AM, *et al.* Connecting genetic risk to disease end points through the human blood plasma proteome. *Nat Commun* 2017; **8**: 14357.

6 Yao C, Chen G, Song C, *et al.* Genome-wide mapping of plasma protein QTLs identifies putatively causal genes and pathways for cardiovascular disease. *Nat Commun* 2018; **9**: 3268.

7 Emilsson V, Ilkov M, Lamb JR, *et al.* Co-regulatory networks of human serum proteins link genetics to disease. *Science* 2018; published online Aug 2. DOI:10.1126/science.aaq1327.

8 Võsa U, Claringbould A, Westra H-J, *et al.* Unraveling the polygenic architecture of complex traits using blood eQTL metaanalysis. 2018; published online Oct 19. DOI:10.1101/447367.

9 Aguet F, Barbeira AN, Bonazzola R, *et al.* The GTEx Consortium atlas of genetic regulatory effects across human tissues. 2019; published online Oct 3. DOI:10.1101/787903.

10 Giambartolomei C, Vukcevic D, Schadt EE, *et al.* Bayesian test for colocalisation between pairs of genetic association studies using summary statistics. *PLoS Genet* 2014; **10**: e1004383.

11 Zheng J, Haberland V, Baird D, *et al.* Phenome-wide Mendelian randomization mapping the influence of the plasma proteome on complex diseases. *Nat Genet* 2020; **52**: 1122–31.

12 Hemani G, Zheng J, Elsworth B, *et al.* The MR-Base platform supports systematic causal inference across the human phenome. *Elife* 2018; **7**. DOI:10.7554/eLife.34408.

13 Wheeler E, Leong A, Liu C-T, *et al.* Impact of common genetic determinants of Hemoglobin A1c on type 2 diabetes risk and diagnosis in ancestrally diverse populations: A transethnic genome-wide meta-analysis. *PLoS Med* 2017; **14**: e1002383.

14 Davies G, Lam M, Harris SE, *et al.* Study of 300,486 individuals identifies 148 independent genetic loci influencing general cognitive function. *Nat Commun* 2018; **9**: 2098.

15 Rees JMB, Foley CN, Burgess S. Factorial Mendelian randomization: using genetic variants to assess interactions. *Int J Epidemiol* 2020; **49**: 1147–58.

16 Campbell JM, Stephenson MD, de Courten B, Chapman I, Bellman SM, Aromataris E. Metformin Use Associated with Reduced Risk of Dementia in Patients with Diabetes: A Systematic Review and Meta-Analysis. *J Alzheimers Dis* 2018; **65**: 1225–36.

17 Hemani G, Bowden J, Davey Smith G. Evaluating the potential role of pleiotropy in Mendelian randomization studies. *Hum Mol Genet* 2018; **27**: R195–208.

18 Schmidt AF, Swerdlow DI, Holmes MV, *et al.* PCSK9 genetic variants and risk of type 2 diabetes: a mendelian randomisation study. *The Lancet Diabetes & Endocrinology* 2017; **5**: 97–105.

19 Davies NM, Holmes MV, Davey Smith G. Reading Mendelian randomisation studies: a guide, glossary, and checklist for clinicians. *BMJ* 2018; **362**: k601.

Supplementary Figures

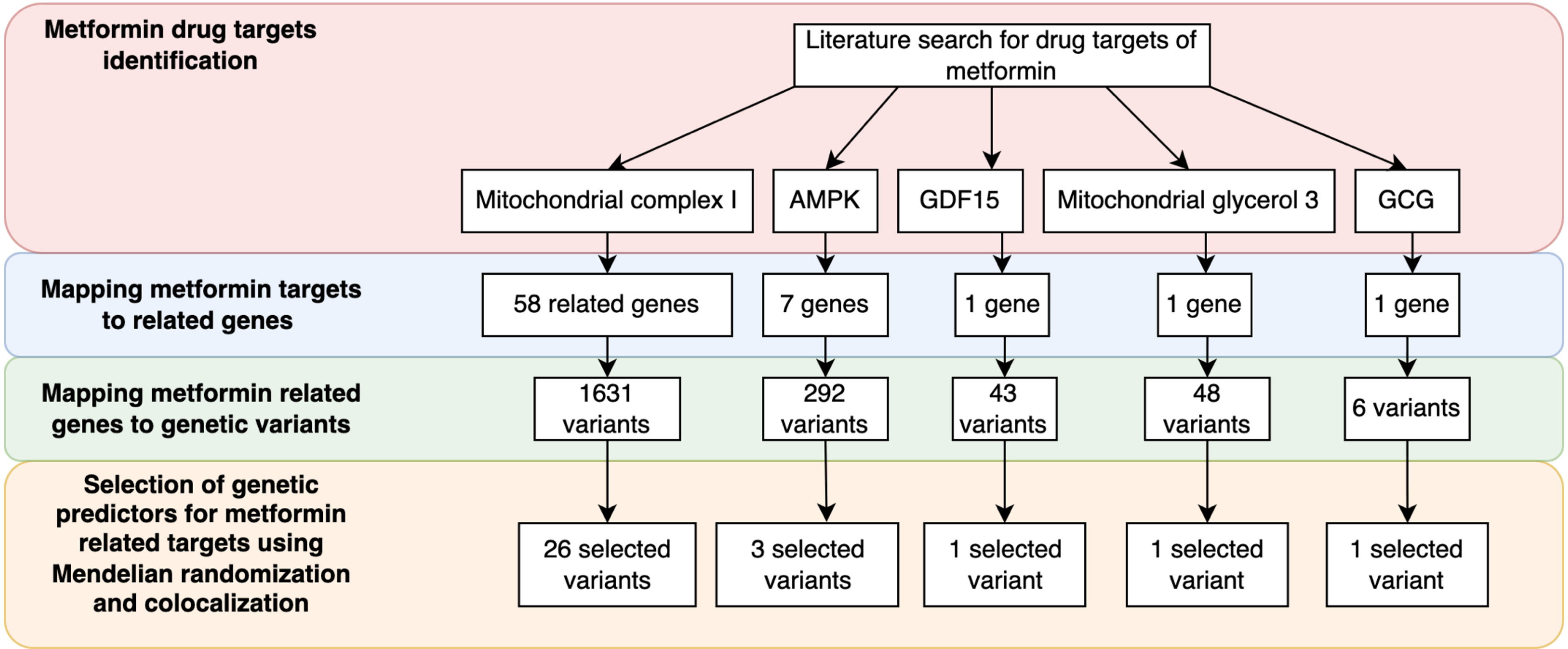

ESM Figure 1. **Diagram of instrument selection of metformin targets.** The selected genetic predictors for the five metformin related targets were selected based on Mendelian randomization and colocalization evidence and listed in **ESM Table 7**.

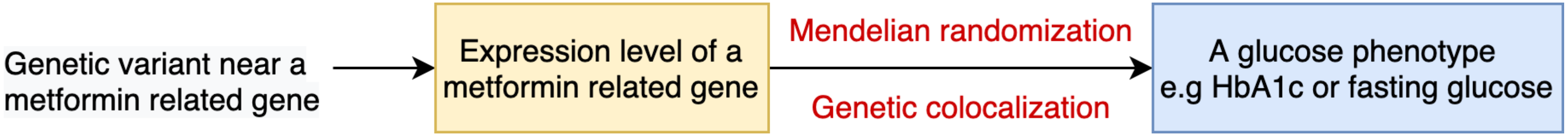

ESM Figure 2. The model for selection of genetic predictors for metformin-related targets and genes.

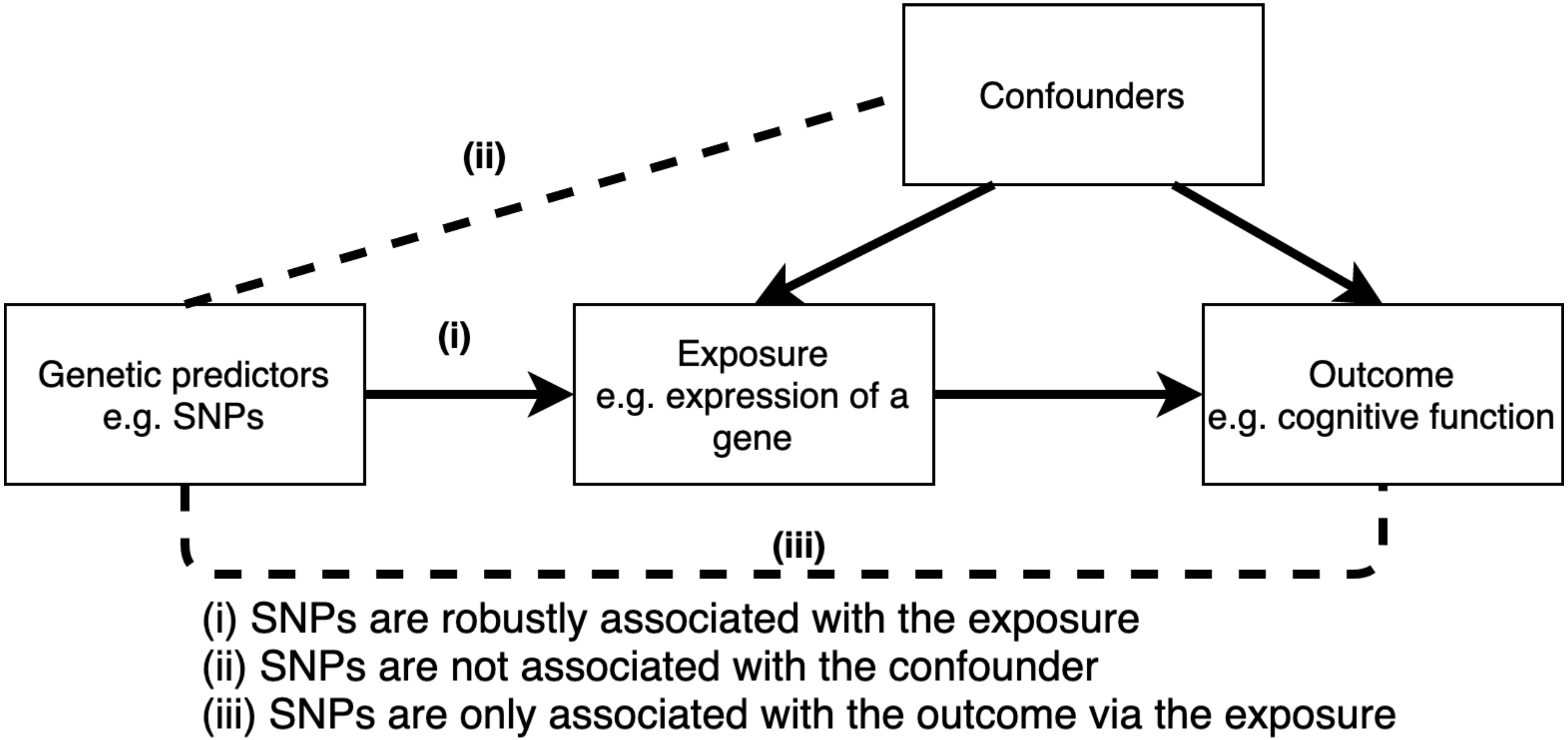

ESM Figure 3. Three core assumptions for Mendelian randomization.

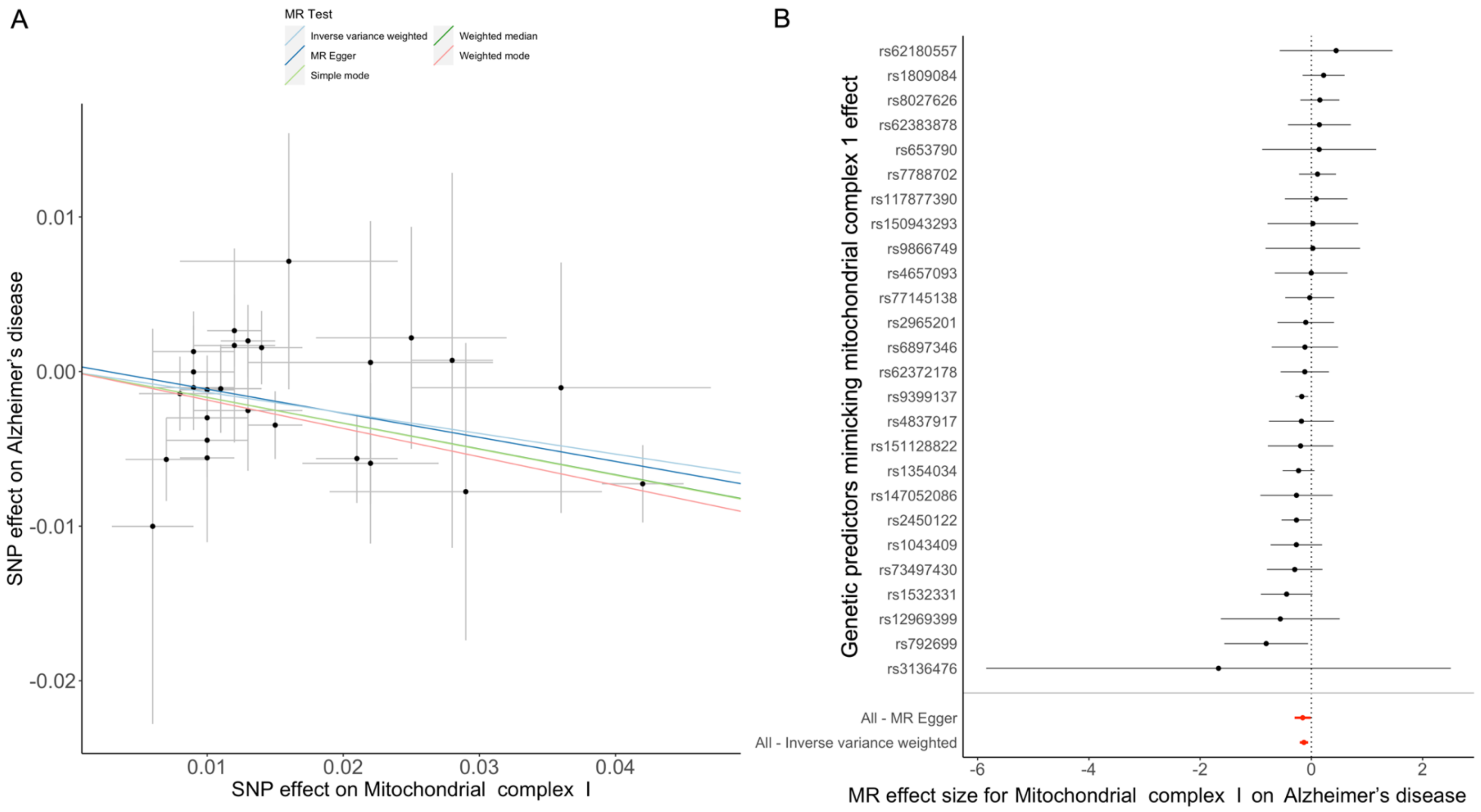

ESM Figure 4. Scatter and forest plots for the mitochondrial complex 1-dependent HbA_{1c} effect of metformin on Alzheimer's disease. (A) scatter plot for the effect of HbA_{1c} lowering via mitochondrial complex 1 on Alzheimer's disease: the slope of the five MR methods agreed very well, which suggested the MR estimates were robust against different MR assumptions; (B) forest plot of single variant Mendelian randomization estimates: this plot suggested that the effect estimates of each genetic predictors on Alzheimer's disease were quite consistent (with overlapped confidence intervals), therefore, the overall MR effect estimate was not driven by any single genetic predictor. Notation: AD in the plot means Alzheimer's disease.

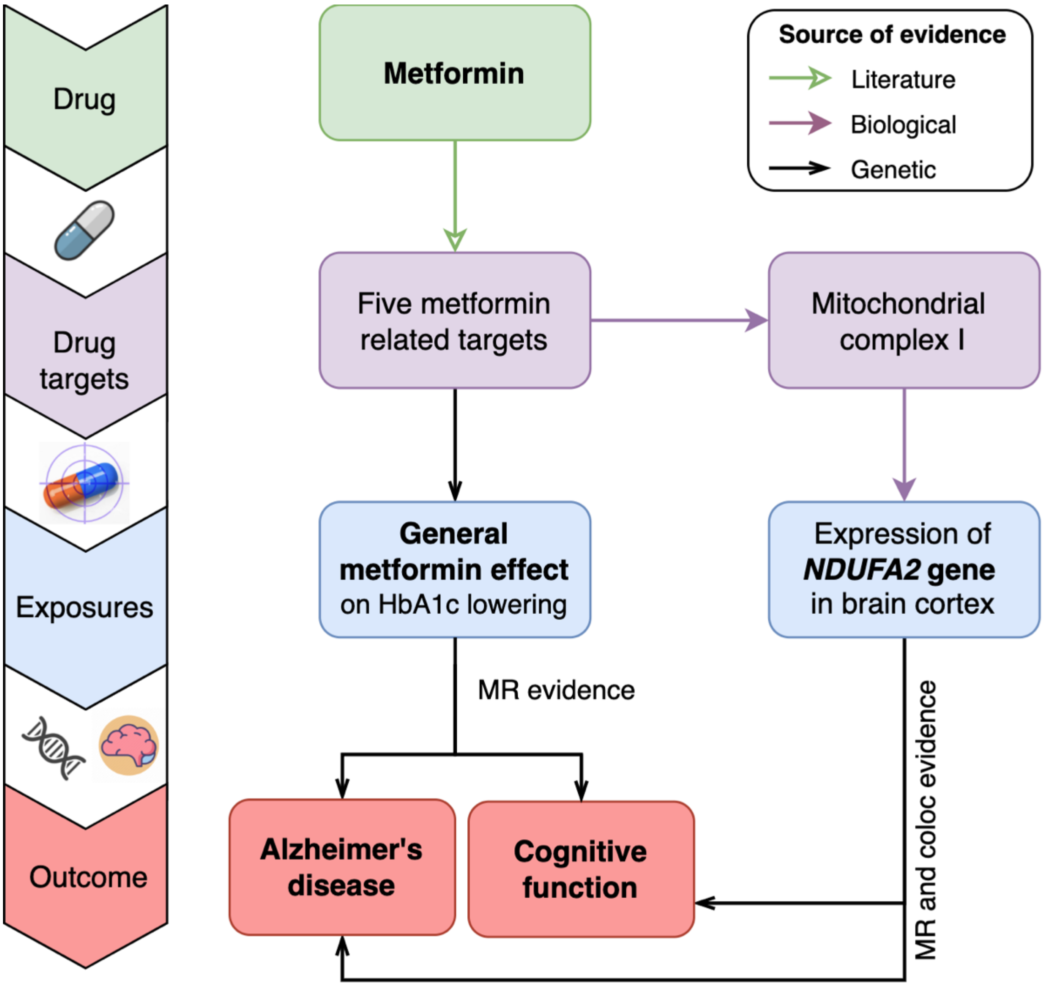

ESM Figure 5. Causal atlas between metformin, brain volume and cognition using genetic evidence. This plot summarized the causal atlas been constructed in this study. For the five components contains in this atlas, we listed the source of evidence linking each pair of components in different array styles, which including literature, biological and genetic evidence. The main causal link (left hand side) refers to the causal effect of metformin's HbA_{1c} lowering effect on Alzheimer's disease and cognitive function. right hand side refers to the putative causal effect of expression of a mitochondrial-related gene, *NDUFA2*, on Alzheimer's disease and cognitive function. Notation: MR refers to Mendelian randomization; coloc refers to colocalisation.
